# 3D Simulation Analysis of Central Shunt in Patient-Specific Hemodynamics: Effects of Varying Degree of Pulmonary Artery Stenosis and Shunt Diameters

**DOI:** 10.1155/2020/4720908

**Published:** 2020-02-14

**Authors:** Jiawei Liu, Haiyun Yuan, Neichuan Zhang, Xiangyu Chen, Chengbin Zhou, Meiping Huang, Qifei Jian, Jian Zhuang

**Affiliations:** ^1^School of Mechanical and Automotive Engineering, South China University of Technology, Guangzhou 510640, Guangdong, China; ^2^Department of Cardiac Surgery, Guangdong Cardiovascular Institute, Guangdong Provincial Key Laboratory of South China Structural Heart Disease, Guangdong Provincial People's Hospital, Guangzhou, China; ^3^Department of Catheterization Lab, Guangdong Cardiovascular Institute, Guangdong Provincial Key Laboratory of South China Structural Heart Disease, Guangdong Provincial People's Hospital, Guangzhou, China

## Abstract

The objective of this study was to compare the effects of different shunt diameters and pulmonary artery (PA) stenosis grades on the hemodynamics of central shunts to determine an optimal surgical plan and improve the long-term outcomes of the operation. A 3D anatomical model was reconstructed based on the patient's clinical CT data. 3D computational fluid dynamics models were built with varying degrees of stenosis (the stenosis ratio *α* was represented by the ratio of blood flow through the main pulmonary artery to cardiac output, ranging from 0 to 30%; the smaller the value of *α*, the more severe the pulmonary artery stenosis) and varying shunt diameters (3, 3.5, 4, 4.5, and 5 mm). Our results show that the asymmetry of pulmonary artery flow increased with increasing shunt diameter and *α*, which will be more conducive to the development of the left pulmonary artery. Additionally, the pulmonary-to-systemic flow ratio (*Q*_P_/*Q*_S_) increases with the shunt diameter and *α*, and all the values exceed 1. When the shunt diameter is 3 mm and *α* = 0%, *Q*_P_/*Q*_S_ reaches the minimum value of 1.01, and the oxygen delivery reaches the maximum value of 205.19 ml/min. However, increasing shunt diameter and *α* is beneficial to reduced power loss and smoother PA flow. In short, for patients with severe PA stenosis (*α* is small), a larger-diameter shunt may be preferred. Conversely, when the degree of PA stenosis is moderate, a smaller shunt diameter can be considered.

## 1. Introduction

For newborns diagnosed with pulmonary artery (PA) atresia or critical stenosis leading to insufficient pulmonary blood flow, a systemic-to-pulmonary shunt (SPS) is a palliative surgical option for establishing a shunt between the PA and the aorta when the development of PA fails to meet the requirements of corrective surgery [1, 2]. This approach can increase blood flow to promote the development of PA and its branches and improve hypoxia, providing conditions for total corrective surgery. However, for patients with PA stenosis, there is no consistent conclusion as to whether to preserve additional pulmonary blood flow (APBF) [3]. It seems reasonable to close the APBF during the central shunt (CS) procedure for most of the special patients because the degree of stenosis is progressive. There is an unstable factor that makes it difficult for surgeons to choose the appropriate shunt size. On the other hand, studies have shown that APBF complements blood supply to the PAs, which results in better systemic arterial oxygen saturation and healthier PA growth [4, 5]. Studies have shown that abnormal hemodynamic factors could lead a series of complications such as thrombosis [6]. At the same time, hemodynamics has strong geometric sensitivity [7, 8]. Hemodynamics parameters such as LPR/RPA flow split, power loss, and so forth will be greatly affected by shunt diameters and pulmonary artery stenosis. Studying the hemodynamics of the central shunt (CS) benefits the improvement of postoperative recovery for patients with an aorta-pulmonary shunt. With this in mind, we study the effects of different degrees of PA stenosis and shunt diameters on hemodynamics of CS.

The postoperative performance of the patient depends on the blood flow balance of the systemic and pulmonary circulation, resulting in maximum oxygen delivery to the tissues. Meanwhile, congestive heart failure, thrombosis, and total power loss should also be considered. Laganà et al. [7, 9] compared the coronary and pulmonary blood flow dynamics of central shunt (CS) and modified Blalock Taussig shunts (MBTS). Piskin et al. [10] studied the hemodynamics of different shunt configurations and diameters in patients with PA atresia. D'Souza et al. [8] used in vitro methods to evaluate the pathophysiologic hemodynamics of a PA stenosis with varying degrees of severity. These studies focused on the hemodynamics of PA atresia or an idealized geometric model but failed to describe local fluid dynamics and the influence of shunt diameter and different degrees of PA stenosis.

For patients born with functional PA stenosis, the degree of stenosis is progressive, which will affect the hemodynamic performance of CS, thereby affecting the quality of life and the risk of long-term complications. To address this, we (1) sought to quantify PA stenosis severity using the ratio (*α*) of blood flow through the main pulmonary artery to cardiac output, with *α* ranging from 0 to 30% and (2) chose common shunts with 3, 3.5, 4, 4.5, and 5 mm shunt diameters under different *α* ratios to assess the impact of these changes on the hemodynamics of the connection using computational fluid dynamics.

## 2. Materials and Methods

### 2.1. Patient Data and 3D Reconstruction

A four-year-old male patient born with pulmonary artery stenosis with ventricular septal defect was retrospectively selected as our research object. The patient information is shown in Table 1. Written informed consent was obtained from the patient's relatives for using clinical data in the study.

**Figure 1:**
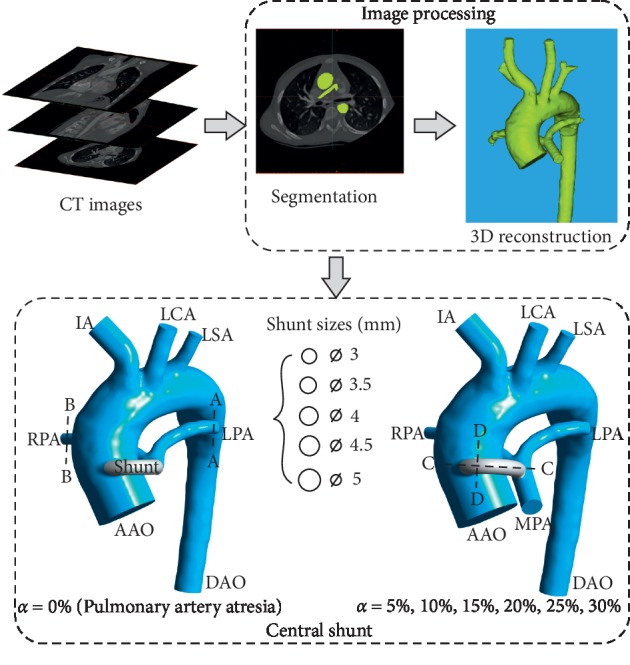
The reconstruction process of the 3D anatomies of the pulmonary artery stenosis patient. Only the 4 mm diameter shunt is shown in the figure, but other sizes configurations (3, 3.5, 4.5, and 5 mm) are provided in scale for comparison and the angle and position of anastomosis remain unchanged. The 3D model on the LEFT represents pulmonary artery atresia (*α* = 0%) and the RIGHT shows pulmonary artery stenosis (*α* = 5%, 10%, 15%, 20%, 25%, 30%). Sections A-A, B-B, C-C, and D-D indicate the cross sections of LPA, RPA, and the shunt, respectively.

Three-dimensional anatomies were reconstructed from preoperative 351 CT images of the patient, including the ascending aorta (AAO), main pulmonary artery (MPA), innominate artery (IA), left carotid artery (LCA), left subclavian artery (LSA), descending aorta (DAO), left pulmonary artery (LPA), and right pulmonary artery (RPA), as shown in Figure 1.

### 2.2. Different Shunt Cases and Mesh Convergence Analysis

In our previous study, we found that the blood flowing into the MPA gradually decreases as the severity of pulmonary artery stenosis increases, and the ratio of blood flow in MPA to cardiac output (CO) was used to define the degree of pulmonary artery stenosis with little effect on results [11]. Thus, in the present study, we analyzed the ratio of blood flow in the MPA to cardiac output in the 55 patients and PA stenosis ratio *α* was used to represent the stenosis severity. The smaller the value of *α*, the more severe the PA stenosis.

(1) $$\begin{array}{c} \alpha =\frac{Q_{\text{MPA}}}{\text{CO}}, \end{array}$$

where *Q*_MPA_ and CO indicate the flow rate of MPA and cardiac output, respectively.

For the shunt model, shunt diameters of 3, 3.5, 4, 4.5, and 5 mm are used. According to the varying levels of stenosis severity, the stenosis ratio *α* can be set to 0% (representing pulmonary artery atresia), 5%, 10%, 15%, 20%, 25%, and 30%, resulting in a total of 35 (5 × 7) simulation cases, as presented in Figure 1.

**Figure 2:**
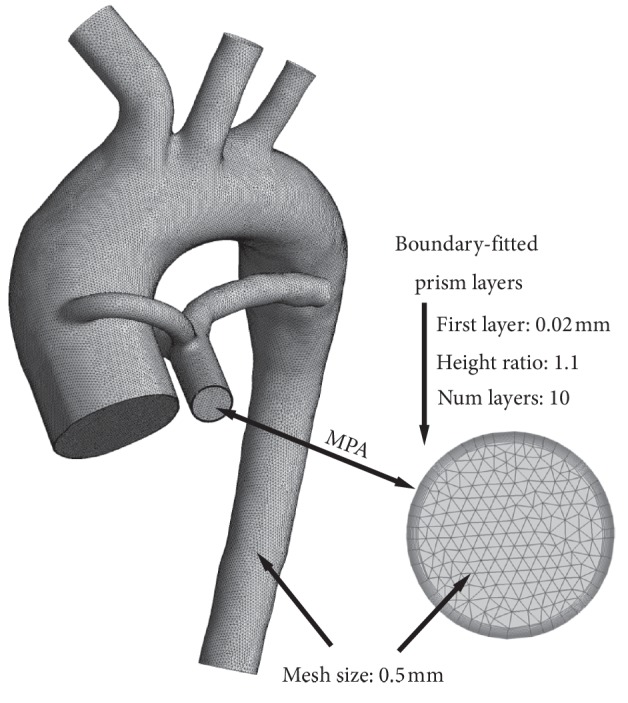
Mesh information.

Five different element sizes were selected to analyze mesh density sensitivity: 0.3 mm mesh size (number of elements: 14,886,800), 0.5 mm mesh size (number of elements: 3,723,041), 0.7 mm mesh size (number of elements: 1,550,800), 0.9 mm mesh size (number of elements: 819,039), and 1.1 mm mesh size (number of elements: 491,068). The types of elements used in all meshes were hexahedral (boundary-fitted prism layers) and tetrahedral elements, ensuring that the mesh quality of all meshes was higher than 0.4. In addition, the boundary-fitted prism layer sizes were set as 1.1 for the height ratio and 10 for the number of layers. A mesh density sensitivity analysis was followed based on achieving a relative difference of less than 0.1% variation in PA flow. Finally, the 0.5 mm mesh size was selected for meshing the whole domain (Figure 2), and this mesh size was used in all simulations.

### 2.3. Boundary Conditions

We have resistance boundary conditions imposed at the outlet vessels of IA, LCA, LSA, DAO, LPA, and RPA to represent the downstream systemic vasculature. The patient-specific resistance values utilized in this study are obtained by matching physiological flow distribution and mean aortic pressure to the appropriate range [7, 12, 13]. They are 2383, 4792, 4260, 1152, 131, and 131 MPa · s · m^−3^ for IA, LCA, LSA, DAO, LPA, and RPA, respectively. Di Molfetta et al. [14] provide the measured data and the reasonable range of mean pressure in arterial systemic and arterial pulmonary. The simulation result of pressure in our study was fall within a reasonable range. Moreover, the difference between simulation pressure in arterial systemic and our patient-specific clinic pressure in arterial systemic was less than 5%. Therefore, the resistance boundary conditions of our study should be reasonable. Although pulmonary vascular resistance (PVR) had a significant effect on pulmonary blood flow [12], the purpose of our study was to investigate the effect of shunt diameter and degree of pulmonary artery stenosis on hemodynamics. Additionally, the resistance (*R*) of downstream systemic vasculature was mainly related to the naive size of vessel [15]. Therefore, the PVR in our study was considered the same as with the change of the shunt diameter or *α*. The porous zone was used to simulate the resistance of downstream systemic vasculature in our study. The viscous resistance of porous zone at the outlet is set to be isotropic and identical. The equation of viscous resistance (*X*) is as follows:

(2) $$\begin{array}{c} X=\frac{R\cdot A}{\mu \cdot l}, \end{array}$$

where *R*, *A*, *μ*, and *l* indicate the resistance value of outlets, the area of outlets, the blood viscosity, and the length of porous zone, respectively.

**Figure 3:**
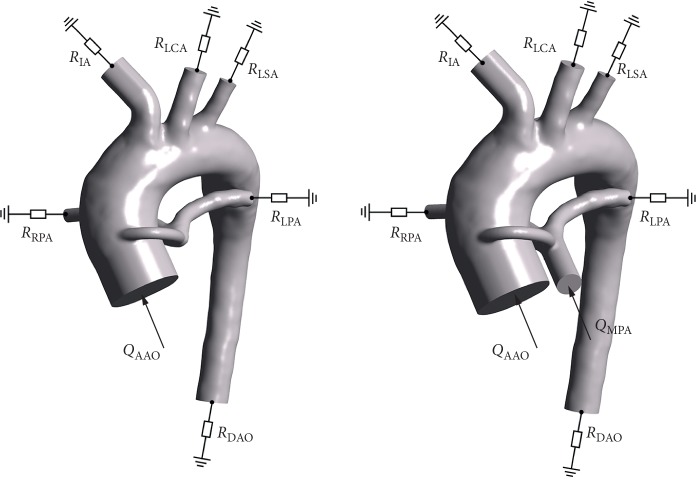
Schematic of resistance boundary conditions. RIA, RLCA, RLSA, RDAO, RLPA, and RRPA represent the downstream vascular resistance of IA, LCA, LSA, DAO, LPA, and RPA, respectively. QAAO and QMPA represent the flow rate of AAO and MPA, respectively.

In our previous study [11], we found that changes in cardiac output had little effect on our results. Therefore, we only consider the effect of shunt diameter on CO, ignoring the effect of PA stenosis severity on CO in the present study. According to Laganà et al. [7], we can set CO of 3, 3.5, 4, 4.5, and 5 mm shunt diameters as 2.122, 2.303, 2.458, 2.630, and 2.798 L/min, respectively. The simulation setup and boundary conditions are summarized in Figure 3.

### 2.4. Computational Fluid Dynamics

Assume that blood is an incompressible Newtonian fluid with a density of *ρ*=1060 kg/m^3^ and viscosity of *μ*=0.005 Pa · s [7, 9, 13]. For all shunt cases, the maximum value of Reynolds numbers is approximately 4500. Therefore, the Shear Stress Transport *k* − *ω* turbulence model is employed in all cases for a coherent treatment of the flows exhibiting Reynolds numbers encompassing the transitional regime [16, 17]. Benim et al. [18] showed that the SST model can accurately accommodate such transitional effects compared to some alternative standard turbulence models. Although the rigid CFD model may have some influence on the results, studies have shown that the difference between the rigid and FSI of the simulation results was within a reasonable range for the hemodynamic parameters [19, 20]. Therefore, 3D domains were assumed to be rigid walled in our study. At the same time, conservation of mass was verified for all cases, and the total difference between inlet flow and outlet flow is less than 10^−8^ kg/s.

### 2.5. Hemodynamic Parameter

Studies have shown that power loss has a great impact on patient exercise performance, especially in patients with congenital heart disease [21–23]. At the same time, power loss (PL) is also a significant hemodynamic parameter used to evaluate the surgical effect. Thus, we define the relative power loss (RPL) to accurately describe the power consumption of different shunt cases.

(3) $$\begin{array}{c} \text{PL}=\underset{\text{in}}{\sum }\left(p+\frac{1}{2}\rho v^{2}\right)Q-\underset{\text{out}}{\sum }\left(p+\frac{1}{2}\rho v^{2}\right)Q,\\ \text{RPL}=\frac{\text{PL}}{\sum_{\text{in}}\left(p+\left(1/2\right)\rho v^{2}\right)}\times 100\%, \end{array}$$

where *p*, *ρ*, *v*, and *Q* indicate the static pressure, density of blood, mean velocity, and volumetric flow rate, respectively.

An important purpose of SPS is to promote the symmetric development of the PA without causing congestive heart failure [8], which is evaluated by LPA/RPA flow split (*Q*_LPA/RPA_).

(4) $$\begin{array}{c} Q_{\text{LPA}/\text{RPA}}=\frac{Q_{\text{LPA}}}{Q_{\text{RPA}}}. \end{array}$$

Another purpose of SPS is to draw off the blood from the aorta into the lungs for oxygenation, thereby avoiding hypoxemia. The optimal situation is to achieve an equitable distribution of blood flow between systemic and pulmonary circulation, thereby maximizing oxygen delivery to the body. Studies have shown that the ratio of pulmonary-to-systemic blood flow (*Q*_P_/*Q*_S_) has a significant effect on oxygen delivery (DO_2_) [24, 25]. Additionally, studies in experimental animals with congenital heart disease have demonstrated that *Q*_P_/*Q*_S_ is associated with oxygen delivery [26–28]. The two equations are as follows:

(5) $$\begin{array}{c} Q_{\text{S}}\cdot C_{\text{art}}-Q_{\text{S}}\cdot C_{\text{ven}}={\text{C}\dot{\text{V}}\text{O}}_{2},\\ Q_{\text{P}}\cdot C_{\text{art}}+{\text{S}\dot{\text{V}}\text{O}}_{2}=Q_{\text{P}}\cdot C_{\text{pv}}, \end{array}$$

where *C*_art_, *C*_ven_, and *C*_pv_ are the O_2_ contents of the systemic arteries, systemic veins, and pulmonary veins, respectively. ${\text{C}\dot{\text{V}}\text{O}}_{2}$ and ${\text{S}\dot{\text{V}}\text{O}}_{2}$ indicate the whole-body oxygen consumption and oxygen uptake in the lungs, respectively.

We assume that the oxygen uptake is equal to the oxygen consumption (${\text{S}\dot{\text{V}}\text{O}}_{2}={\text{C}\dot{\text{V}}\text{O}}_{2}$). Therefore, the oxygen delivery is calculated as

(6) $$\begin{array}{c} {\text{DO}}_{2}=\frac{1}{1+Q_{\text{P}}/Q_{\text{S}}}\text{CO}\cdot {\text{C}}_{\text{pv}}-\frac{1}{Q_{\text{P}}/Q_{\text{S}}}{\text{C}\dot{\text{V}}\text{O}}_{2}, \end{array}$$

where CO is the cardiac output (CO=*Q*_S_+*Q*_P_). The pulmonary venous flow oxygen saturation is defined as 96%, so *C*_pv_ = 0.2112 (mL oxygen/mL blood) [29] and whole-body oxygen consumption ${\text{C}\dot{\text{V}}\text{O}}_{2}=18\;\text{mL}\;\text{oxygen}/\min$ [30].

## 3. Results

### 3.1. *Q*_LPA/RPA_ and *Q*_P_/*Q*_S_

**Figure 4:**
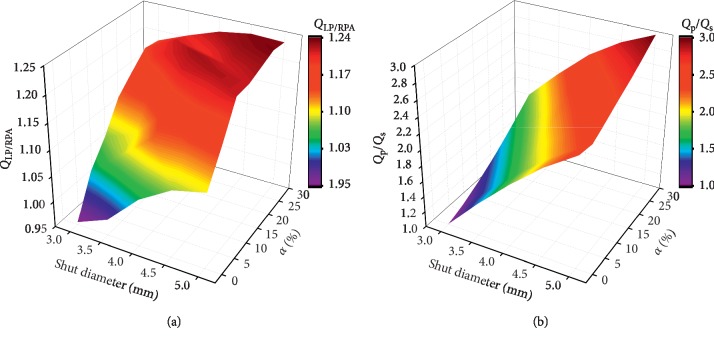
Cloud map of *Q*_LPA/RPA_ and *Q*_P_/*Q*_S_ changing with the shunt diameters and stenosis ratio *α*.

Postoperative pulmonary perfusion is considered to be the primary parameter for patients with PA stenosis, while the LPA/RPA flow split is related to the symmetrical development of pulmonary arteries.  Figure 4(a) depicts the cloud map of *Q*_LPA/RPA_ changing with the shunt diameters and stenosis ratio *α*. These results suggest that the augmentation in shunt diameter or *a* led to an increase in *Q*_LPA/RPA_. However, when *α* remains unchanged, the choice of larger shunt diameters increases the total pulmonary flow but does not alter the PA split significantly. *α* is one of the main factors affecting *Q*_LPA/RPA_. In the case of 3, 3.5, 4, 4.5, and 5 mm shunt diameters, the difference in *Q*_LPA/RPA_ reached 23.6%, 22.7%, 17.5%, 14.3%, and 12.5% when *a* was changed from 0% to 30%. In general, in most cases, *Q*_LPA/RPA_ > 1; that is, the blood flow in LPA is higher than the blood flow in RPA, which will be more conducive to the development of LPA. The only two exceptions are the cases at 3 and 3.5 mm shunt diameters (*α* = 0%), *Q*_LPA/RPA_ of which are 0.955 and 0.981, respectively. The results also show that when *α* = 0%, *Q*_LPA/RPA_ is closer to 1, which is beneficial to the symmetrical development of PA.

Figure 4(b) shows the effect of shunt diameter and *a* on *Q*_P_/*Q*_S_. The results show that *Q*_P_/*Q*_S_ increases monotonically with the shunt diameter and *α*. Different from *Q*_LPA/RPA_ in Figure 4(a), *Q*_P_/*Q*_S_ is more greatly affected by the shunt diameter than *α*. For shunt diameter sizes of 3, 3.5, 4, 4.5, and 5 mm, the *Q*_P_/*Q*_S_ ratio ranges are 1.01–1.80, 1.42–2.18, 1.79–2.53, 2.13–2.78, and 2.40–2.96, respectively. These results suggest that *Q*_P_/*Q*_S_ > 1 in almost all cases. However, *Q*_P_/*Q*_S_ is strongly influenced by PA resistance. When PA resistance is high, *Q*_P_/*Q*_S_ may be less than 1 for smaller shunt diameters.

### 3.2. Oxygen Delivery DO_2_

**Figure 5:**
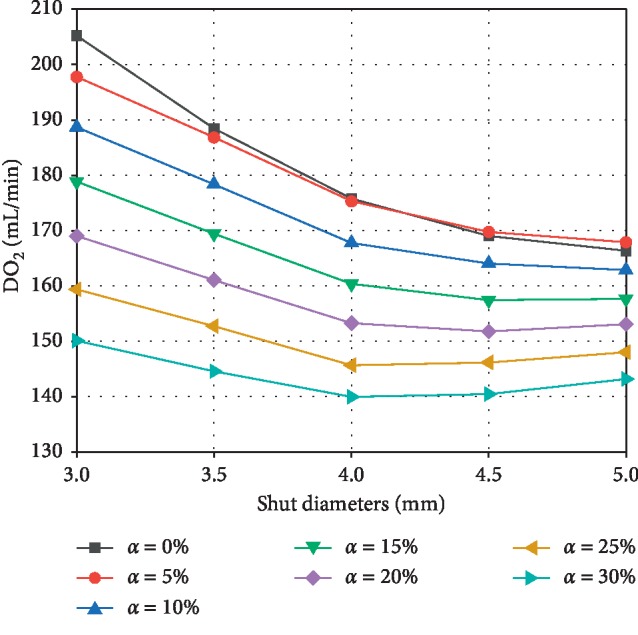
Effect of shunt diameter on oxygen delivery (DO_2_) under different *α*.

Figure 5 depicts the variation of the oxygen delivery (DO_2_) with the shunt diameter under different *α*. DO_2_ is a function of *Q*_P_/*Q*_S_ and CO (in equation (6)). These results indicate that DO_2_ decreases with increasing shunt diameter and *α*. This trend is associated with increasing *Q*_P_/*Q*_S_ (see Section 3.1). However, with the increase of the shunt diameter and *α*, the declining trend of the DO_2_ curve gradually becomes smooth. Especially when *α* = 25% or 30%, DO_2_ increases slightly as shunt diameter increases from 4 mm to 5 mm. This could be partially explained by the increase in CO. The results also show that the effect of shunt diameter on oxygen delivery decreases as *α* increases. When shunt diameter is changed from 3.0 to 5.0 mm, DO_2_ decreases by 18.93% under *α* = 0%, and DO_2_ decreases by only 4.61% under *α* = 30%.

### 3.3. Power Loss and Relative Power Loss

**Figure 6:**
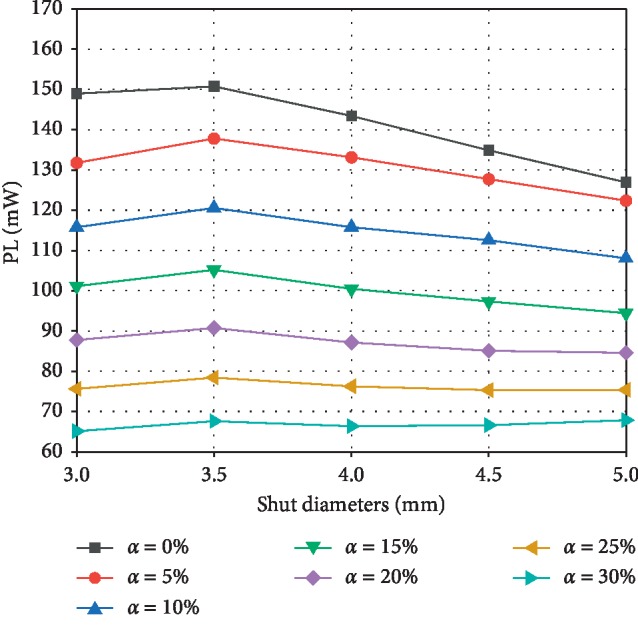
Power loss at different shunt diameters and PA stenosis ratios *α*.

**Figure 7:**
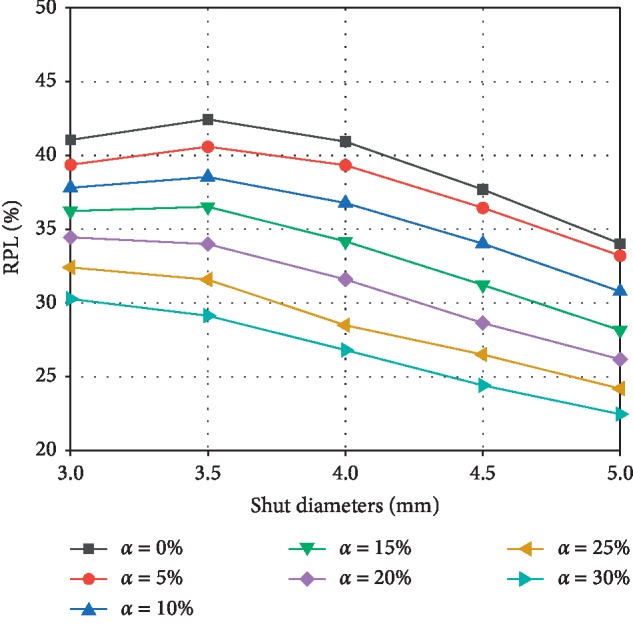
The relative power loss changing with stenosis ratio *α* under different shunt diameters.

Studies have shown that increased power loss in the physiological cycle can have an adverse impact on the quality of life and the risk of long-term complications [31, 32]. Figures 6 and 7 depict the change in power loss (PL) and relative power loss (RPL) with shunt diameter and *α*. The results show that under the same shunt diameter, PL and RPL decrease with increasing *α*; that is, the more severe the PA stenosis, the greater the power loss. As *α* increases from 0% to 30%, the PL ranges of shunt diameters of 3, 3.5, 4, 4.5, and 5 mm are 148.9–65.1, 150.7–67.6, 143.4–66.4, 134.9–66.6, and 126.9–67.8 mW, respectively. For shunt diameters of 3, 3.5, 4, 4.5, and 5 mm, the RPL ranges are 41.0–30.3%, 42.4–29.1%, 40.9–26.8%, 37.7–24.4%, and 34.0–22.5%, respectively. These results also indicate that increasing the shunt diameter is beneficial to the reduction of RPL at the same *a*. The only exception is when *α* ≤ 15%, the RPL of 3 mm is smaller than 3.5 mm diameter.

### 3.4. 3D Flow Streamlines and Flow Field Distribution

**Figure 8:**
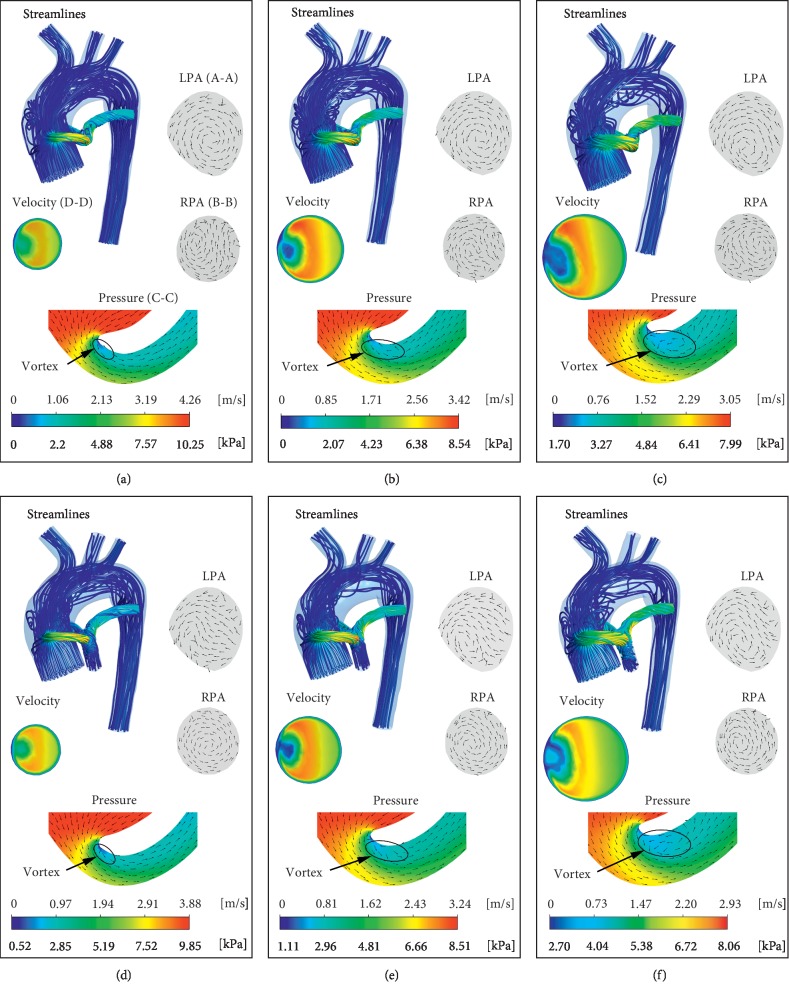
3D blood flow streamlines under the 3, 4, and 5 mm shunt diameter with *α* of 0% and 5%. Velocity vector of LPA and RPA represents vector distribution of sections A-A and B-B (Figure 1), respectively. The velocity distribution at the cross section (section D-D in Figure 1) of the shunt is shown. The pressure distribution and velocity vector at the cross section of the shunt (section C-C in Figure 1) are also shown in the figure: (a) shunt = 3 mm *α* = 15%, (b) shunt = 4 mm *α* = 15%, (c) shunt = 5 mm *α* = 15%, (d) shunt = 3 mm *α* = 30%, (e) shunt = 4 mm *α* = 30%, and (f) shunt = 5 mm *α* = 30%.

**Figure 9:**
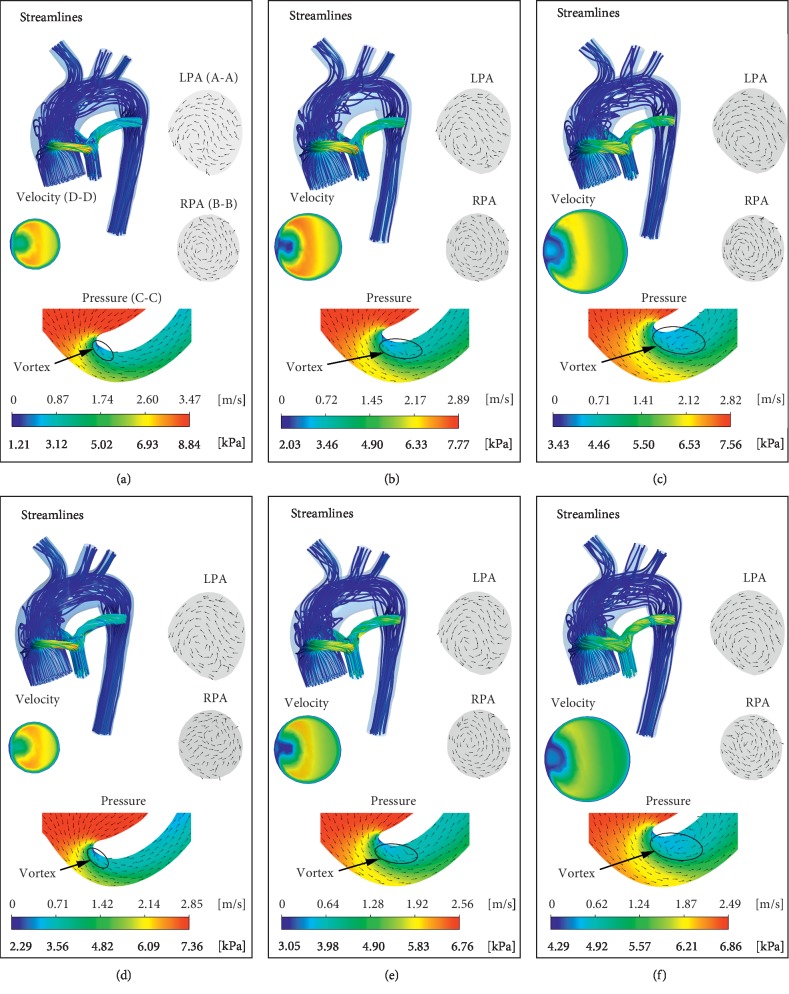
3D blood flow streamlines under the 3, 4, and 5 mm shunt diameter with *α* of 15% and 30%. Velocity vector of LPA and RPA represents vector distribution of sections A-A and B-B (Figure 1), respectively. The velocity distribution at the cross section (section D-D in Figure 1) of the shunt is shown. The pressure distribution and velocity vector at the cross section of the shunt (section C-C in Figure 1) are also shown in the figure: (a) shunt = 3 mm *α* = 15%, (b) shunt = 4 mm *α* = 15%, (c) shunt = 5 mm *α* = 15%, (d) shunt = 3 mm *α* = 30%, (e) shunt = 4 mm *α* = 30%, and (f) shunt = 5 mm *α* = 30%.

Abnormal hemodynamics are associated with complex flow structure, shunt diameters, and pulmonary artery stenosis ratio (*α*). Figures 8 and 9 depict the flow fields at 3, 4, and 5 mm shunt diameters with *α* values of 0% (pulmonary artery atresia), 5%, 15%, and 30%. Under the same shunt diameter, the maximum velocity in the 3D domain gradually decreased with increasing *α*. When *α* remains unchanged, the flow velocity will also decrease with increasing shunt diameter. When *a* is not equal to 0, as *α* gradually increases, the blood flow in the MPA gradually increases. As expected, the blood from the shunt flows into the PA and gradually mixes with the blood in the MPA, and the blood streamlines become smoother. When *α* = 0, swirl is prone to be generated in LPA, but when *α* is not equal to 0, the RPA produces high vorticity regions compared to LPA. Swirl formation contributes to the power loss and can partially explain the trends presented in Section 3.3.

In all cases, the velocity distribution at different cross sections (Section D-D) of the shunt is similar. The high-velocity regions are mainly distributed outside of the shunt, while the low-velocity areas are mainly distributed inside the shunt. From the pressure distribution in the cross section (Section C-C) of the shunts, the pressure gradient at the proximal anastomosis of the shunts is large, which is consistent with the results of Pennati et al. [33]. Moreover, there is a low-pressure region close to the inner wall at the proximal anastomosis, which is highly related to the “T-shape” topology of shunt anastomosis. As we expected, the vortex is formed at the low-pressure region. The area of the vortex region is closely related to the shunt diameter but has little to do with the stenosis ratio *α*. As the shunt diameter increases, the vortex area gradually increases.

## 4. Discussion

In the CS cycle, *Q*_LPA/RPA_, *Q*_P_/*Q*_S_, power loss, and hemodynamic deficiencies can significantly affect postoperative performance, impeding the child's ability to play normally with peers. In this study, based on the actual situation of the patient, different shunt diameters and PA stenosis ratios *α* were studied to assess the impact of these changes on the hemodynamics of the connection using computational fluid dynamics.

Survival after such an operation is very dependent on the balance between systemic and pulmonary blood flows, which in turn is highly dependent on the fluid dynamics through the shunt [7]. Hence, numerical modeling can help the surgeon in the choosing optimal choice of shunt size based on the individual patient. D'Souza et al. [8] showed that the RPA/LPA flow is not symmetric in all shunt configurations. In particular, the CS configuration causes nonsymmetric pulmonary artery flow in favor of LPA for all shunt sizes studied, which is consistent with our study. Additionally, our study suggests that the asymmetry of PA flow increased with increasing shunt diameter and *α*, which will be more conducive to the development of the LPA. The only exception is when *α* = 0% (PA atresia), *Q*_LPA/RPA_∼1, which is beneficial to the symmetrical development of PA. Additionally, *α* is the main factor affecting *Q*_LPA/RPA_, which may be related to the geometry of PA in the patient. For example, *Q*_LPA/RPA_ is related to the difference between the angle branching of the RPA and the LPA from main pulmonary artery. With more right-angle branching of the LPA than the RPA from main pulmonary artery, the blood flow is prone to flow to LPA from MPA. Studies have shown that DO_2_ and saturation are directly related to the calculated *Q*_P_/*Q*_S_ ratio [19, 24, 25]. Our results show that the ratio of *Q*_P_/*Q*_S_ increases with the shunt diameter and *α*, and all the values are greater than 1. Optimal oxygen delivery is achieved when balanced pulmonary and systemic perfusion is established, namely, when *Q*_P_/*Q*_S_∼1 [13]and high values are associated with low DO_2_, which is only partially compensated by increases in CO [24]. Our results demonstrate that when the shunt diameter is 3 mm and the APBF is closed (*α* = 0%), *Q*_P_/*Q*_S_ reaches the minimum value of 1.01, and DO_2_ reaches the maximum value of 205.19 ml/min. Bakir et al. [12] constructed an in vitro setup of mBT shunt in normal anatomical conditions and investigated the effects of different flow rates and pulmonary vascular resistances (PVR) on shunt flow. Their results show that the pulmonary flow is associate with the PVR. The value of PVR varies among patients, thereby leading to different *Q*_P_/*Q*_S_ and DO_2_ values under the same shunt diameter. However, our research can still show the trend of *Q*_P_/*Q*_S_ and DO_2_; that is, a smaller shunt diameter and *α* are conducive to oxygen delivery.

Independent of the type of disease, high energy dissipation always impacts the function of the heart, which, together with the peripheral vasculature, strives to overcome additional resistance to meet the function of the circulatory system, thereby resulting in chronic heart failure as a secondary disease [34]. As *α* increases, a significant decrease in power loss is observed for all shunt cases. The decrease is dramatically high, reaching above 50% when *α* is changed from 0% to 30%. Compared with *α*, the shunt diameter has less influence on power loss. The decrease is no more than 15% when the diameter is changed from 3.0 mm to 5.0 mm. Therefore, in terms of energy, choosing a larger shunt diameter helps to reduce the burden on the heart for patients with certain PA stenosis.

Hemodynamic characteristics are closely related to complex flow structures. Our results show that when *α* increases gradually, that is, with greater proportion of APBF, the PA flow gradually becomes smooth. This is consistent with the trend of energy loss. Due to the “T-shape” topology of the anastomosis, the low-pressure zone at the anastomosis of the shunt is also a low-velocity region, which will lead to abnormal hemodynamic factors such as vortex, reflux, and flow separation that are dangerous signals of thrombosis [6]. Flow visualization analysis shows that as the shunt diameter increases, the prominent recirculation and stagnation zones become larger. Characteristically, such zones have low shear stress promoting platelet activation, aggregation, and thrombosis, especially within the lumen of the synthetic graft [35, 36]. At the same time, the increase in shunt diameter may lead to insufficient blood supply to the upper body and congestive heart failure caused by overflowing to the lungs. Therefore, the above factors should be taken into account in the selection of the diameter to help surgeons select the appropriate shunt diameter.

### 4.1. Limitations

We recognize several limitations of our study. First, the calculation model is a rigid-walled CFD model, which does not account for arterial compliance within its domain. Second, this study only considers the effect of shunt diameter on cardiac output and ignores the effect of PA stenosis on cardiac output. For given values of CO and ${\text{C}\dot{\text{V}}\text{O}}_{2}$, the analysis can predict DO_2_ by calculating the *Q*_P_/*Q*_S_ ratio. However, it cannot predict the whole-body response to a change in one variable. For example, in the case of exercise, ${\text{C}\dot{\text{V}}\text{O}}_{2}$ and CO are increased, which will affect DO_2_. Third, the predictions discussed in the present study are therefore most applicable in the short term after surgical recovery, particularly given that we have not modeled long-term growth in the pulmonary arteries.

## 5. Conclusion

There is no golden rule for shunt diameter size for patients with varying degrees of pulmonary artery stenosis as these parameters should be decided based on the characteristics of the individual patient. There is a need for compromise among PA flow split, oxygen delivery, optimal power loss, and smooth PA flow. Compared to larger shunt diameters, a smaller shunt diameter is beneficial to oxygen delivery and less prone to lead to pulmonary artery overflow and congestive heart failure; however, a smaller diameter means greater power loss, which will increase the burden of the heart. As such, for the same shunt diameter, different stenosis ratios *α* will also affect hemodynamic parameters such as DO_2_ and power loss. An increase in *α* is beneficial for reducing power loss, but it reduces DO_2_ and affects the symmetric growth of the pulmonary artery. In general, a larger shunt diameter may be preferred for patients with severe pulmonary artery stenosis (*α* is small). Conversely, when the degree of pulmonary artery stenosis is moderate, a smaller shunt diameter can be considered.

## Figures and Tables

**Table 1:** The patient information and clinically measured hemodynamic data.

Item	Details
Sex	Male
Age (year)	4
Weight (kg)	11
McGoon	0.94
Nakata index	46.21
Aortic systolic pressure (mmHg)	105
Aortic diastolic pressure (mmHg)	59

## Data Availability

The data used to support the findings of this study are available from the corresponding author upon request.
